# Opportunities and barriers for smaller portions in food service: lessons from marketing and behavioral economics

**DOI:** 10.1038/ijo.2014.85

**Published:** 2014-07-25

**Authors:** J Riis

**Affiliations:** 1 Department of Marketing, The Wharton School, University of Pennsylvania, Philadelphia, PA, USA

## Abstract

This paper uses the frameworks and evidence from marketing and behavioral economics to highlight the opportunities and barriers for portion control in food service environments. Applying Kahneman's ‘thinking fast and slow' concepts, it describes 10 strategies that can be effective in ‘tricking' the consumer's fast cognitive system to make better decisions and in triggering the slow cognitive system to help prevent the fast system from making bad decisions. These strategies include shrinking defaults, elongating packages, increasing the visibility of small portions, offering more mixed virtue options, adding more small sizes, offering ‘right-sized' standard portions, using meaningful size labels, adopting linear pricing, using temporal landmarks to push smaller portions and facilitating pre-commitment. For each of these strategies, I discuss the specific cost and revenue barriers that a food service operator would face if the strategy were adopted.

Around the world, but especially in America, consumers are eating more energy in meals away from the home than in the past years. Restaurant meals have become larger, and we eat more of them.^1,2^ With increased energy consumption closely associated with the rise in obesity,^3^ many authors have suggested portion control as an important means to reduce the trend.^4^ In the current paper, I will use frameworks and evidence from marketing and behavioral economics to highlight the opportunities and barriers for portion control in food service environments. The opportunities and barriers will be considered from the perspective of both the customer and the food service operator.

## The customer

### Thinking fast and slow

Why do consumers tend to overeat in the first place? If one takes the view that overeating is rational, then there is nothing to explain—the value of the short-term pleasure of overeating would be considered to outweigh the long-term costs. But this rational story is hard to defend in light of the facts that many consumers wish that they did not overeat,^5^ wish that they were not overweight and frequently fail to lose weight after paying out of pocket costs for diet and exercise products and services. It is also hard to defend in light of the very high long-run monetary and quality-of-life costs associated with overeating.

Behavioral economics, in contrast to classical economics, offers a systematic way in which to think about apparent irrationalities in consumer decision making. It starts with the ideas that there are limitations in the degree to which consumers can (a) exert control over their own behavior and (b) gather and process relevant information. These limitations exist because the human mind is the result of a long evolutionary process that builds on ancient perceptual and emotional neurological mechanisms. The oldest such mechanisms are essentially automatic. They lead to decisions that are very fast, effortless, uncontrolled, emotional and often unconscious. Newer and more uniquely human mechanisms also operate within our brains. These reflective mechanisms, by contrast, lead to decisions that are slow, effortful, controlled, deductive and generally involve self-awareness. The automatic system is involved when we speak our native language. The reflective system is responsible when we try to learn a foreign language.^6^ Kahneman^7,8^ has used the phrase ‘thinking fast and slow' to characterize the interaction of these automatic and reflective systems in the human mind.

Decisions about which foods to eat, and how much to eat, rely largely on the fast, automatic processes. The behavioral economics framework offers some strategies, described in the section 'Portion reduction opportunities', that may be effective in ‘tricking' the fast system to make better decisions and in triggering the slow system to help prevent the fast system from making bad decisions.

### Kinds of customers

While obesity is prevalent in all demographics, and oversized portions are seen in all types of restaurants, there is considerable variance. Obesity is more common among low- and middle-income populations, and oversized portions are more commonly seen in the quick serve and casual dining restaurants they frequent. For that reason, most of this discussion will use America's largest casual dining chain, Applebee's, as its example, and its traditional counterpart in the quick serve category, McDonald's.

Demographics aside, there are important psychographic (or attitudinal) differences between customers. These distinctions can be made in fine gradations, but I will use a simple binary categorization for discussion purposes—‘eager' versus ‘reluctant' customers. An eager customer is one who recognizes that her eating habits are not optimal. A Pew survey recently found that 60% of Americans know that they overeat.^5^ Solutions for this segment will be different than for the ‘reluctant' segment that is not interested in change.

Children will of course generally fall into the reluctant segment. Children are importantly different in the food service domain in another way—they are usually not paying customers, so while they need to be kept happy, it is a different relationship than with a paying adult customer.

## The food service operation

Before describing the portion-size opportunities available to food service operators, it is necessary to list the major risks to profitability, both on the revenue side and on the cost side. Public health researchers and regulators need to be aware of these barriers if they are to engage operators in dialogue and collaboration.

### Revenue barriers

Since ‘value', or ‘value for money' is one of the main consumer benefits of large portions, food service operators would rightly worry that value perception would be hurt if portion sizes are reduced. This is not only true as chains compete with each other, but it is also true as the industry competes with home-cooked meals. If all chains had to reduce portion sizes, and consumers saw less ‘value' in eating out, the industry as a whole could suffer. So it is no wonder that the industry generally opposes portion-size mandates.

Value perception could be addressed by lowering the prices if smaller portions are being served, but of course lowering the prices in itself is a significant and direct revenue risk.

Relatedly, although portion control itself is a potential consumer benefit, and could potentially increase the revenue if consumers actually eat out more frequently as the risk of overeating declines, chains may wonder if they can really compete on this basis. ‘Less is more' is probably not a compelling marketing message to most consumers.

Even if smaller portions did have some appeal, and did not damage value perception, they could affect consumer value in a more direct way, through reduced meal enjoyment. If consumers were frequently worried that they were not going to leave a restaurant satiated (or as satiated as they would like to be) they may enjoy the outing less, and they may crave the meals less. Similarly, consumers may just enjoy overeating, even if they know it is not good for them. Once portions make overeating more difficult, excitement about a meal may decrease. Reduced excitement about the dining experience is a major concern for repeat business.

All of these considerations can lead to erosion of a chain's brand, reduced revenue and ultimately reduced profitability.

### Cost barriers

Cost is the other side of the profitability equation, and firms could rightly worry that reducing portion sizes could actually increase the costs.

Reducing portions sizes should generally reduce the cost of goods sold (COGS) but it may not do so in a linear manner. Unit costs could increase if the reduction is large enough that it affects the volume discount the operator gets from its supplier.

Firms might also decide that portion-size reductions will require positioning the chain more on quality and less on value. To increase quality, the operator may need to pay a higher price to buy higher-quality basic commodities. If they do increase quality, however, this would presumably be offset by an increase in revenue if the cost is passed onto the consumer with a price increase, but it is not clear exactly how this would balance out. It is possible that profitability would be hurt.

Relatedly, if smaller portions simply mean reducing the volume, there could be savings in inventory cost, but there could also be inventory increases if the smaller-portion meals have different ingredients than the large-portion meals.

If new products are developed to accommodate smaller portions, then research and development costs will be incurred by the operator. If smaller portions need to be higher in quality, or need to use different ingredients to be more filling, or even if they just require new packaging or plating, research and development costs could be involved.

Smaller portions can also add operational complexity if new sizes are introduced. Operational complexity is associated with increased labor costs, and possibly costs associated with changes to standard operations. Cooks and servers may need to be trained not to over-portion, and training requires both management and labor time, and this involves additional cost.

If menus change, or if smaller portions will be served more often, or if they need to be promoted by operation staff, then there will be communication costs. These costs will involve devising messaging that will appeal to consumers, and to internal constituencies to enhance organizational alignment. Any change in a large organization requires extensive internal communication.

### Kinds of food service operations

Different kinds of food service operations will have different kinds of revenue and cost pressures, different kinds of customers, and therefore different opportunities for reducing portion sizes.

Cafeterias, both school and workplace, have more latitude in terms of customer satisfaction because their customers typically have less choice about where to eat. At the same time, cost pressure is substantial because prices generally have to be kept very low.

Restaurants that are high-end (that is, ‘fine dining') will tend to have more wealthy customers who are more likely to be eager with respect to healthy eating. And they will be under less pressure to keep portions large as their customers are less concerned about value. They can also spend more on staff to prepare portions that are very efficient in terms of calories.

Even within the casual dining category, it is easier to innovate with portion-size meals targeted at eager, wealthier customers. Applebee's has a series of dishes that have less than 550 calories, aimed squarely at their eager customers. Darden (owners of Red Lobster, Olive Garden and others) recently opened a chain called Seasons 52, which serves only ‘small plates' with less than 475 calories. Generally, when a customer group is more concerned about cuisine, preparation, experimentation, and so on, they will be less concerned about large portions. Casual dining chains are constantly innovating their menus and looking for opportunities to be taste leaders. New and interesting preparations that people might see on a TV food network will not have to be served in large portions because the target customer is more driven by curiosity and adventure, than by value.

## Portion reduction opportunities

For all of the opportunities listed here, there is evidence from marketing and behavioral economics that they could increase the likelihood that consumers will choose smaller portions. At the same time, there are revenue and cost barriers to each of them. It is beyond the scope of this paper to quantify these revenues and costs (see Dittmer and Keefe^9^ for a detailed treatment), but I will offer suggestions as to which of the barriers are likely to be the biggest, and in which situations.

I will suggest 10 different opportunities. Each consists of a menu or service change that operators could use to move consumers in the smaller directions. The opportunities described first generally rely more on ‘tricking' the fast, automatic thinking system. The opportunities described later tend to rely more on ‘triggering' the slow, reflective thinking system.

### Change the default to a smaller portion

Default effects can be very powerful and indeed their existence is one of the major sources of support for the behavioral economics paradigm. One of the most widely cited default findings is that organ donation rates vary greatly between different countries, seemingly mostly based on whether or not organ donation cards have an opt-in or opt-out box. In Sweden, where citizens were defaulted into organ donation (but could opt out by signing their driver's license), organ donation rates were 85%. In culturally similar Denmark, where citizens were defaulted out (but could sign to opt in), organ donation rates were only 4%.^10^ If defaults have such powerful effects in deeply personal and important decisions like organ donation, they ought to have an effect in more routine decisions like food choice as well.

And indeed they do. In 2006, the Disney Corporation began serving apples as the default in all kids' meals instead of French fries. Disney reports that the ‘overwhelming majority of families' accept the default option rather than choosing an alternative. It is worth noting, however, that a meal at Disney is not a routine meal, and no one has yet systematically measured what would happen if a large operator decided to make smaller meal portions its default.

So we can ask the question, what would happen if Applebee's simply reduced the standard portion of French fries served, or reduced the size of some of its highly caloric dishes, like hamburgers? Even a reduction of 10% could save many calories.

The beauty of defaults is that most people do not notice when defaults change, making it a fast brain strategy. This means that both eager and reluctant customers will be served smaller portions, probably without noticing. The problem, however, is that some customers would notice. Some might get angry and stop returning to the chain. And some may only come to notice slowly that the meals have gotten smaller but that prices have remained the same. For these customers, the value of the meal may decline gradually. If somehow the story of smaller defaults gets into the press, there is more risk of value and brand erosion.

The smaller default could certainly be used more effectively for energy-dense items at school and workplace cafeterias, where value perception is not as much of a concern. There is also a lot of room for further experimentation with smaller defaults with children's meals at restaurants, but even these opportunities are not without risk.

### Elongate packages and servings of energy-dense items

If smaller default portions are used, they can be packaged and presented in such a way as to limit the extent to which they are noticed. Research by Chandon and Ordabayeva^11^ has shown that people are much more likely to notice downsizes when a package changes in only one dimension. In one experiment, the researchers first showed consumers a small serving of movie popcorn in a cube-shaped 24 oz bucket. When the bucket was enlarged in all three dimensions into a 110-oz cube, consumers estimated the volume to be only 54 oz. When, instead, the bucket was enlarged in only one dimension, into an elongated 110-oz box, consumers still underestimated the volume, but they were much closer, with an average estimate of 81 oz. Chandon and Ordabayeva conclude that less healthy products should be served in ‘elongated' packages to give the perception of large size, without actually serving such large quantities. This is a fast-brain strategy because customers do not have to stop and reflect on a choice to take a smaller portion—fast and automatic brain processes lead them to perceive a smaller portion as being large enough. Accordingly, it can be effective with both eager and reluctant consumers.

This packaging change is a real opportunity, but it is not without revenue and cost barriers. In some cases, especially for take-out food, the elongating of the package may not preserve heat as well, or it may fit differently into bags, or visually customers may not be used to it. Research and development efforts could test these issues, but such efforts will not be costless.

For in-store dining, this elongation approach could work with smaller defaults. If Applebee's, for example, reduced its standard French fry portion, fewer people would presumably notice if they managed to plate the French fries in an elongated manner, thus reducing the possible revenue risk. Servers would have to be trained to serve fries in this manner, and they may need to be careful about how this is explained to guests, if guests ever asked. For operations where value erosion is a major concern in reducing portions, the costs of changing packaging or plating may be worth absorbing.

### Make smaller portions easier to find

It is a basic truth of retailing that if a particular product is given a more prominent location in a store^12,13^ or on a menu,^14^ then it is more likely to be chosen. In supermarkets, the effectiveness of this strategy accounts for millions of dollars that manufacturers spend annually in slotting fees. The same principle applies in food service settings, although few studies have demonstrated the magnitude of the effect. In one large study, however, at a hospital cafeteria, Thorndike *et al.*^15^ increased the sales of bottled water by 30% over 3 months by simply placing bottles of water in baskets beside each food station in the cafeteria.

The same idea should work for portion sizes. If smaller portioned dishes get more prominent placement on a menu, or if smaller portions get more prominent placement within a cafeteria, they will be more likely to be chosen. Value erosion can be reduced if larger portions are still visible and available to consumers who really do want them. So there is some opportunity here.

The cost lies mainly in the opportunity cost of placing less-valued items on prime menu or shelf real estate. Food service operations always want to make their ‘best-selling' items most visible and available. Hiding products that consumers want will involve some risk. This risk can most easily be managed in workplace and school cafeterias.

### Change default to smaller portion of less-healthy foods and add healthier ones

Another way to reduce the default sizes of less-healthy foods is to serve them with additional portions of complementary healthy items. For example, some restaurants offer customers French fries, salad, or half-and-half. Half-and-half could be made the new default at places like Applebee's. McDonald's has experimented with something similar in its Happy Meals, replacing its traditional small French fries with a small serving of French fries (100 calories) plus apple slices.

The advantages of this approach are that it allows customers to satisfy their craving for French fries while reducing energy intake, and not undermining value perception (because the smaller portion of fries is made up with something else). Still, risks remain.

If consumers do not like the virtue item (salad or apples) they may throw it out, not only leading to unnecessary waste, but also leading the customer to perceive a value loss. Even if they eat the virtue item, many consumers may not enjoy it very much, at least compared to the French fries, leading to reduced satisfaction. This tradeoff is one that many ‘eager' health customers will like, but reluctant customers will be more resistant to. So the opportunity is biggest with chains that have a large eager segment.

The approach will not work for all menu categories. For example, it will not work straightforwardly for things that cannot be mixed or put on the same plate, like beverages. Still, beverages can be served in separate glasses or bottles. In addition, some blends may work better than previously thought. The Mushroom Council is now working with food operators to market a blended burger that is 60% beef and 40% mushroom. If something like that became the default, higher-calorie beef portions would effectively be reduced.

The main cost barrier in most of these mixed offerings is that in most cases it adds to product and operational costs. Generally, the operator will need more of the healthier items, which tend to be more expensive (although perhaps not in the case of beef versus mushrooms), and adding two items to a side dish instead of one involves operational costs in cooking, serving and communicating.

Still this is one of the best opportunities because it limits the major threat of value erosion. And operational innovation can reduce the cost of most complicated processes. The question is will any operators invest the research and development dollars to make this happen?

### Add more small size options to the menu

There are still some places where small sizes are not really available, so operators do not really know if consumers would order them. Even if the small size is offered at only a modestly lower price, many consumers may still choose it.

Some firms may reason that they will just have a large size and if consumers want less they will just eat less. That is probably not true. When more food is put on the plate, people eat more.^16^

Vermeer (this volume) describes two studies in which adding smaller portions to a menu did result in a substantial number of consumers choosing the small portion. She does not report on the effects on restaurant revenue. Even if the small size was not priced much lower, it would probably still have a negative effect on the bottom line. Sometimes, consumers may buy a smaller size and use the ‘savings' to buy a dessert or an additional item. So the only way that the addition of smaller sizes can help both profit and health is if the consumer does make an additional spend, and the additional spend is on a low-calorie item.

Even if the addition of the smaller sizes does lead to additional spends at the restaurant, it does still involve the addition of another product, which adds operational complexity. On the other hand, a chain may decide that the presence of smaller sizes is good for revenue because it helps the brand and consumers will be happy that they have more opportunities to manage their portions. This could make the operational complexity worth the trouble.

Some chains have built their entire value proposition around smaller portions. Seasons 52, mentioned earlier, only offers items under 475 calories. In addition to the health benefits of smaller portions, this tapas style of eating allows the consumer to choose more items, perhaps increasing the variety. The risk there, from the health point of view, is that increased variety tends to increase total consumption. It would be interesting to know what the average caloric load is of typical ‘total meals' served at Seasons 52. Do customers actually eat less there than they would at other chains with different menu structures?

While the efforts of Seasons 52 are laudable, it is targeting a wealthier, older consumer who is more likely to be an ‘eager' health consumer in the sense used earlier. But that of course is what makes it a business decision, not a public health decision. The hope, from a public health point of view, is that the Seasons 52 concept will trickle down to a broader customer base.

### Offer to ‘right size' standard portions

The 2004 film Supersize Me put McDonald's in the obesity bull's eye when it highlighted the chain's service policy of asking customers if they would like to ‘super size' their portions of fries and soda for a small premium. This policy became an icon of the obesity problem, but it also showed just how effective point-of-sale (POS) offers can be.

Schwartz *et al.*^17^ turned the idea around with a POS intervention where customers were explicitly invited to downsize their portion. They conducted a series of experiments at a Chinese food themed quick serve location. On test days, the servers had been trained to ask all customers if they would like to save calories by taking a smaller portion of their (highly caloric) side dishes of fried rice, steamed rice and lo mein. In one experiment, 35% of customers agreed to downsize, and the offer of a nominal discount ($.025 on a $7.00 meal) did not affect the acceptance rate.

The authors argue that many customers at the chain knew that they typically ate too much (cf. Pew Research Center^5^) and were open to the opportunity to put less food on their plate if they were asked at the right time. These eager customers will make a healthier choice if their slow brains are triggered at the right moment.

This approach is not one that most chains will want to take on, since it requires effort to train all of the staff to make the offer, the savings on food may be quite modest (at least in this case), and it is always possible that some customers will object. However, Schwartz *et al.* argue that the phrase ‘right size' could become part of the standard service lexicon, and as guests start to request it operators ought at least be ready to respond. Currently, in many casual and quick serve restaurants, if a customer asks for a smaller-than-usual portion, the server simply does not know what to do. If ‘right sized' became a more widely known concept, some chains may be willing to start with some more subtle promotion around it, for example, using point-of-sale signage, which may be seen as less risky.

### Use meaningful size labels

Calorie labels have received the most attention, and have been most widely implemented in food service operations. Their success has been mixed. While some studies show that they have had a modest effect on purchase behavior, other studies show null results. Either way, they are set to take effect nationwide following a mandate in the affordable care act. Even if they do not affect consumer behavior, there is evidence that they have affected firm behavior.^18^ It is worth noting that calorie labeling has been an expensive endeavor, both for industry, and for regulators.

Until consumers become very good at understanding, adding and keeping track of calories, their use as the main source of information about size is unlikely. Consumers are still likely to make inferences from the size labels given to foods and beverages. Even if a ‘small' portion of fries has 400 calories, consumers will be likely to treat it as a small. Just and Wansink^19^ showed a version of this effect when they served two groups of consumers identical two cup portions of pasta. Consumers ate more pasta when it was labeled ‘regular' size than when it was labeled ‘double-size'.

Schwartz *et al.*^17^ have encouraged the use of a phrase like ‘right-sized', although it may be hard to legislate around that. Even industry self-regulation may be difficult. What is right sized for one person will be different for another. Still, if meal sizes did start being served in round and somewhat standard calorie amounts, something like 500 or 600 calories could become widely understood as the right meal size. With a specific calorie target in mind for meals, consumers may start to make the right tradeoffs to meet that target.

While the ban on oversized portions in NYC is in limbo, there would be other ways to address supersizes. For example, Thorndike *et al.*^20^ found that traffic light labels were very effective in changing consumer behavior in a large hospital cafeteria over a 2-year study. Labeling very large portions with a red label may serve as a reminder to customers that they are probably eating too much. This could work in workplaces but would be a difficult thing to mandate in restaurants, and few would likely take it up.

### Use linear pricing

It is not unusual to see very large unit price differences between small and large items on a menu. At McDonald's for example, 16 ounce small soda might cost $0.99 (i.e., 6.2 cents/ounce) while a 32 ounce large soda might cost $1.49 (i.e., only 4.7 cents/ounce). The small is a terrible ‘deal' compared to the large. If the small had the same unit price as the large, many more people would order it. Some have suggested that linear pricing, whereby unit price must be constant across sizes, be mandated.

This is an interesting possibility, but it is important to note that it would almost certainly have to be mandated. Dobson and Gerstner^21^ showed that non-linear pricing around sizing is very much in the firm's interest, because it costs so relatively little to increase portions. In fact, they argue that small sizes are priced high to discourage price sensitive consumers from buying them, while the relatively low prices of very large portions entice consumers to buy them, and to over-consume as a result.

Customers are so accustomed to these pricing strategies that in a competitive environment it will be very difficult for an individual firm to adopt linear pricing.

### Use temporal landmarks to push small portions

The slow brain is more easily triggered on some days than on others. Dai *et al.*^22^ have found that consumers are more likely to engage in health behaviors on Mondays, on the first day of a month, and immediately after birthdays and national holidays. These days represent breaking points in life and create the experience of a fresh start. The fresh-start experience may trigger the slow brain—on these breakpoint days, Dai *et al.* observe that consumers are more likely to search for the term ‘diet' on Google, attend the gym and enter commitment contracts to achieve various aspirational goals, including saving more money, studying harder and losing weight.

Consumers might also be more open to small-portion messaging on such days. Meatless Mondays have been introduced in various community campaigns. Restaurant chains may find that small-portion promotions (perhaps with discounts) are a good draw for customers on these breakpoint days and may actually generate traffic.

The danger of these promotions is of course the general point that any kind of promotion costs money. It takes marketing dollars to make consumers aware of the promotion, and training dollars to get staff to execute it.

### Allow people to pre-commit to healthy lunch

When consumers make choices for consumption experiences in the future, they are more likely to make more prudent selections than if they are making choices for consumption experiences in the present. For example, people save more money when they are deciding how much to save next year versus this week,^23^ they choose more ‘high-brow' films when they are deciding what to watch two days from now versus tonight,^24^ and they choose more healthy grocery items when the groceries will be delivered in a few days versus tomorrow.^25^

This tendency is a slow-brain process—it is easier to reflect on decisions made for the future because fast-brain cravings are less prominent.^26^ The opportunities to use these slow-brain processes through pre-commitment are rare in food service. People can order food for pickup, but pickup is usually immediate, not a day or two in advance. If people could order tomorrow's lunch at a time when they were not particularly hungry, they may be more likely to order a healthier (and smaller) lunch.

While the opportunity here is large, the barriers are also substantial. Most food service operations are not set up to take pre-orders. While the basic technology exists, it is yet to be implemented.

There are also revenue risks in terms of the consumer experience. Customers may become too ambitious in their pre-ordering, and may end up with items that they are not happy with. They will blame themselves to some extent, but the restaurant may share some of the blame (even if undeserved).

## Conclusions

The list of strategies listed here is not exhaustive. But it does contain some particularly promising approaches that use psychological insights within the fast-brain/slow-brain paradigm, and apply them to food service. Some are unlikely to work without major policy interventions or industry coordination. In this list I would include things like the default change, using meaningful size labels and linear pricing. They could be effective in less-competitive environments like cafeterias, but I expect that a competitive landscape will generally make them challenging.

I think there is more opportunity with package elongation, mixing vice and virtue, right sizing and using temporal landmarks. These are approaches or promotions that chains can try themselves and are, for the most part, not terribly risky.

The opportunities for pre-commitment will continue to grow as more customers order food online. A danger of the proliferation of online technologies is that it makes it even easier for consumers to make impulse food purchases, which are likely to be higher in calories. If the technologies were set up to allow, and even encourage, meal selection for a future time point, impulse (that is, the fast brain) may yield to the slow brain.

As there is more and more pressure on food service operators to serve safer portions, there should be more opportunities to create partnerships with researchers to understand what works best, with which kinds of customers and in which kinds of operations.

Currently, it is difficult to get good estimates of the effect size of any particularly change in practice. There are at least four impediments. First, operators have a lot of intuitions that are very often untested. They know that some customers will object if they try something, but they do not know how many. Second, public health researchers and regulators often do not appreciate all of the barriers (both cost and revenue) that prevent action on the part of operators. Third, there are many customer and operator factors that make for tremendous variation in the likely size and, even direction, of any intervention. Finally, there have been very few carefully controlled field experiments done through independent researchers. If academics better understand the challenges to operators, they may be better able to invite research collaboration.

## Notes

JR has consulted for Compass Group and ConAgra.
